# Bronchoalveolar lavage fluid from preterm infants with chorioamnionitis inhibits alveolar epithelial repair

**DOI:** 10.1186/1465-9921-10-116

**Published:** 2009-11-23

**Authors:** Jasper V Been, Luc JI Zimmermann, Anne Debeer, Nico Kloosterboer, J Freek van Iwaarden

**Affiliations:** 1 Department of Paediatrics, School for Oncology and Developmental Biology (GROW), Maastricht University Medical Centre, PO Box 5800, 6202 AZ Maastricht, the Netherlands; 2 Department of Neonatology, University Hospital Gasthuisberg, Herestraat 49, 3000 Leuven, Belgium

## Abstract

**Background:**

Preterm infants are highly susceptible to lung injury. While both chorioamnionitis and antenatal steroids induce lung maturation, chorioamnionitis is also associated with adverse lung development. We investigated the ability of bronchoalveolar lavage fluid (BALF) from ventilated preterm infants to restore alveolar epithelial integrity after injury *in vitro*, depending on whether or not they were exposed to chorioamnionitis or antenatal steroids. For this purpose, a translational model for alveolar epithelial repair was developed and characterised.

**Methods:**

BALF was added to mechanically wounded monolayers of A549 cells. Wound closure was quantified over time and compared between preterm infants (gestational age < 32 wks) exposed or not exposed to chorioamnionitis and antenatal steroids (≥ 1 dose). Furthermore, keratinocyte growth factor (KGF) and vascular endothelial growth factor (VEGF) were quantified in BALF, and their ability to induce alveolar epithelial repair was evaluated in the model.

**Results:**

On day 0/1, BALF from infants exposed to antenatal steroids significantly increased epithelial repair (40.3 ± 35.5 vs. -6.3 ± 75.0% above control/mg protein), while chorioamnionitis decreased wound-healing capacity of BALF (-2.9 ± 87.1 vs. 40.2 ± 36.9% above control/mg protein). BALF from patients with chorioamnionitis contained less KGF (11 (0-27) vs. 0 (0-4) pg/ml) and less detectable VEGF (66 vs. 95%) on day 0. BALF levels of VEGF and KGF correlated with its ability to induce wound repair. Moreover, KGF stimulated epithelial repair dose-dependently, although the low levels in BALF suggest KGF is not a major modulator of BALF-induced wound repair. VEGF also stimulated alveolar epithelial repair, an effect that was blocked by addition of soluble VEGF receptor-1 (sVEGFr1/Flt-1). However, BALF-induced wound repair was not significantly affected by addition of sVEGFr1.

**Conclusion:**

Antenatal steroids improve the ability of BALF derived from preterm infants to stimulate alveolar epithelial repair *in vitro*. Conversely, chorioamnionitis is associated with decreased wound-healing capacity of BALF. A definite role for KGF and VEGF in either process could not be established. Decreased ability to induce alveolar epithelial repair after injury may contribute to the association between chorioamnionitis and adverse lung development in mechanically ventilated preterm infants.

## Background

Antenatal steroid administration and intrauterine inflammation are two important factors capable of promoting lung maturation before birth. Maternal administration of corticosteroids in case of anticipated preterm delivery enhances structural lung maturation and stimulates surfactant secretion in the fetus, and has become standard of care in current obstetric practice [1,2]. Intrauterine inflammation, represented histopathologically by chorioamnionitis, stimulates lung maturation and decreases the incidence of the respiratory distress syndrome (RDS) [3-6].

Although both chorioamnionitis and antenatal steroids induce lung maturation, a single course of antenatal steroids does not seem to affect longer term lung development while chorioamnionitis does [1,3,4]. Several studies have shown an association between chorioamnionitis and subsequent development of chronic lung disease of prematurity (bronchopulmonary dysplasia; BPD) [3]. Experimental chorioamnionitis in animals, besides inducing lung maturation, also results in a pathological picture of alveolar simplification similar to that of BPD currently seen in preterm infants [4]. This suggests that, although the short term effect appears similar, both entities induce different responses affecting subsequent lung development. The notion that antenatal steroid administration further reduces RDS incidence in preterm infants exposed to intrauterine inflammation, also suggests that both processes exert their effects at least partially through distinct mechanisms [3].

The mechanisms by which the differential effects of antenatal exposure to steroids and inflammation induce lung maturation and affect lung development are incompletely understood. Different effects of both antecedents on pulmonary growth factor expression have been demonstrated [2,4,7]. However, lung development is a dynamic, complex and tightly regulated process involving multiple cell types, effector molecules and interactions. By quantifying biological activity of human lung-derived specimens, all potential modulators present are allowed to conduct their effects. An available model for this purpose is the epithelial wound healing model. After induction of a mechanical defect in cultured epithelial cells, the effect of a modulator on the ability of the epithelium to restore its integrity is evaluated. A translational approach can be made by evaluating the effect of human lung-derived fluid obtained *in vivo *on alveolar wound healing capacity *in vitro *[8]. Using this approach relevant differences between distinct patient groups have been reported [9,10].

This model has not been applied in neonatal pulmonary medicine, while it offers comparison of biological activity of lung-derived fluid between patients that were or were not exposed to either antenatal steroids or chorioamnionitis to evaluate their effects on the ability to restore alveolar epithelial injury. This is of particular interest for chorioamnionitis, since the relationship between chorioamnionitis and adverse lung development has been shown to be predominantly modulated by postnatal injury. Clinical data suggest that chorioamnionitis in itself is not a risk factor for adverse lung development and may actually even be protective, while subsequent postnatal exposure to either sepsis, mechanical ventilation or both highly increases BPD risk [11-14].

We hypothesised that chorioamnionitis reduces the ability of lung-derived fluid to restore alveolar epithelial integrity after injury, while antenatal steroids would have a stimulatory effect. To test this hypothesis, we developed a translational *in vivo-in vitro *model for alveolar epithelial repair. By collecting bronchoalveolar lavage fluid (BALF) at consecutive postnatal time points, we evaluated the time-dependent postnatal effect of both antenatal modulators on BALF biological activity. We further investigated a potential underlying role of two growth factors in the BALF-modulated repair process. Vascular endothelial growth factor (VEGF) is invaluable for normal lung development and is decreased in lung-derived fluid from infants developing BPD [15-18], and in sheep lungs after experimental chorioamnionitis [7]. Keratinocyte growth factor (KGF) is a potent stimulant of alveolar repair after lung injury [19-25] and high KGF in tracheal aspirate fluid (TAF) has been associated with absence of BPD [26]. Importantly, both growth factors have been shown to enhance *in vitro *alveolar epithelial wound healing [27-29].

## Methods

### Patient characteristics and enrolment

Patients were eligible for the study when born before 32 weeks gestational age and ventilated for RDS. Patients were enrolled in the NICUs in the University Hospitals of Maastricht and Leuven. The Medical Ethical Committees of both hospitals approved the study and written parental consent was obtained. Chorioamnionitis was diagnosed histologically when >10 neutrophils per high-power field were present in the chorion or amnion. Steroids were administered to the mother in case of anticipated preterm delivery by giving two intramuscular doses of betamethasone acetate 12 mg 24 hrs apart.

### BALF collection and processing

BALF was performed at postnatal days 0-1, 3-4, and 7 according to a standard procedure. After turning the infant's head to the left, a 6 French suction catheter was inserted through a side port of the Trachcare^® ^closed suctioning system until slight resistance was felt. Then, one ml per kg birth weight of sterile isotonic saline solution was gently infused into the lung. After five seconds, suctioning was performed while slowly retracting the catheter. The procedure was repeated once after which the collected fluid was pooled and placed on ice. After centrifugation for 10 minutes at 4°C and 300 × *g*, the supernatant was collected, aliquoted and stored at -80°C until analysis.

### *In vitro *alveolar epithelial wound healing assay

A549 cells (Sigma-Aldrich, St. Louis, MO, USA) were seeded in 24-well plates (BD, Franklin Lakes NJ, USA; approximately 200.000 cells per well) and grown to confluence at 37°C and a 5% CO_2_-95% air atmosphere in RPMI 1640 medium (Dutch modification; Invitrogen, Carslbad CA, USA) supplied with 10% (v/v) fetal bovine serum (FBS; Greiner Bio-One, Kremsmünster, Austria), 200 μM L-glutamine (L-glut; Invitrogen, Carslbad CA, USA) and 10 U penicillin + 10 μg streptomycin per ml (P/S; Invitrogen, Carslbad CA, USA). A black grid printed on a plastic sheet was attached to the bottom of each plate, positioning a 2 × 2 mm square underneath the centre of each well. Between the lines of each square a rectangular wound was created by gently scratching the monolayer with a sterile 1 ml pipette tip. By visualisation, scratches of poor quality were excluded from further analysis before start of the experiment. After wounding, each well was washed with 1 ml of phosphate-buffered saline (PBS (pH 7.4); Invitrogen, Carslbad CA, USA) to remove cellular debris.

For model validation experiments, effects of FBS and albumin on wound healing were evaluated by adding various concentrations of FBS to RPMI 1640 medium + L-glut + P/S or human serum albumin (A1887, Sigma-Aldrich, St. Louis MO, USA) to control medium (RPMI 1640 + 0.1% (v/v) FBS + L-glut + P/S), using 300 μl medium per well. Various concentrations of BALF diluted in control medium were added to wounded monolayers, using 300 μl medium per well, to evaluate the effect of BALF dilution on wound healing. For this purpose, BALF was obtained from a term newborn ventilated for non-pulmonary reasons.

### Effect of BALF, KGF and VEGF on in vitro alveolar epithelial wound healing

For BALF experiments, BALF was diluted 1:10 in control medium and each well was supplied with 300 μl of this mixture from one BALF specimen after wounding. Control wells were supplied with isotonic saline 1:10 in control medium. All experiments were performed in triplicate and each 24-well plate contained three controls. Moreover, each plate included one positive control containing 10% (v/v) FBS. In additional experiments, various concentrations of human KGF (R&D systems, Minneapolis MN, USA) diluted in control medium were added to wounded monolayers in 24-well plates. VEGF (10 pg/ml; R&D systems, Minneapolis MN, USA), soluble VEGF-receptor 1 (sVEGFr1/Flt1; R&D systems, Minneapolis MN, USA) (6 ng/ml) and BALF (1:10 (v/v)) diluted in control medium were added in distinct experiments, both solitary and combined, to evaluate their effects on alveolar epithelial wound repair. For this purpose BALF from several patients was pooled to obtain a large and uniform sample. Each plate contained three controls and quadruplicates for each growth factor/receptor concentration.

### Assessment of wound healing

Using a Leica DC 300 F camera coupled to a Leica MZ FL III stereomicroscope, each wound was photographed at a magnification of 3.15× directly after scratching (t = 0) and at subsequent time points (6 and 24 hours for BALF experiments). Each well was repeatedly photographed within the central square of the grid to ensure wound surface measurements were performed at a fixed position.

The wounded surface was quantified by tracing the wound edge and calculating the denuded surface area using Image J software. The assessor was blinded for the experimental condition of the photograph. Wound healing was assessed by subtracting the wound area at a given time point from the wound area at time point zero and expressed as the percentage of wound healing of each BALF specimen above the wound healing of the control wells (containing 0.1% FBS (v/v)) on the same culture plate.

### Quantification of total protein, KGF, VEGF, sVEGFr1, and M30

Total protein in BALF was quantified using the NanoOrange^® ^protein quantitation kit (Molecular Probes, Eugene, OR, USA) according to the manufacturer's instructions. KGF, VEGF and sVEGFr1 in BALF were quantified by ELISA (R&D systems, Minneapolis MN) according to the manufacturer's instructions, in half-area 96-well ELISA plates (Greiner Bio-One, Kremsmünster, Austria). To assess the effect of BALF on induction of apoptosis in A549 cells, M30 (CK18Asp396-NE; a soluble marker of epithelial cell apoptosis) was quantified in the cell culture supernatant at the end of the wound healing experiment (t = 24) using ELISA (Peviva AB, Bromma, Sweden). For this purpose, triplicates from the culture experiments were pooled to represent a single patient sample.

### Statistics

Continuous data are expressed as mean ± standard deviation if normally distributed, and median plus interquartile range otherwise. Differences between groups were tested using student t-test and ANOVA with post-hoc Bonferroni correction for comparison of two or three groups, respectively. Mann-Whitney U test was used for comparison of non-parametric data and dichotomous variables were tested using χ^2^-test. Correlations were determined using Pearson's correlation coefficient. A test result of p < 0.05 was considered significant (two-tailed). All analyses were performed using SPSS 15.0 software.

## Results

### FBS stimulates *in vitro *alveolar epithelial wound healing

**Figure 1:**
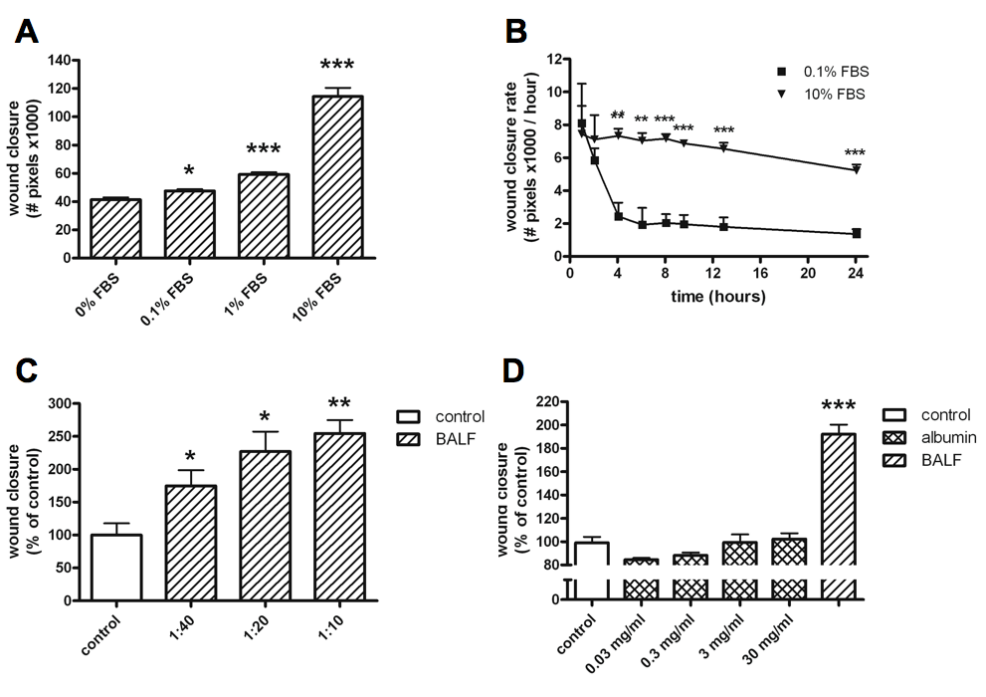
**Characterisation of the alveolar epithelial wound repair model**. **A**. Effect of addition of various concentrations of fetal bovine serum (FBS) to culture medium on *in vitro *alveolar epithelial wound healing (A; t = 24 hours). Bars represent mean wound closure + SEM (number of pixels ×1000) of triplicate experiments (*p < 0.05; ***p < 0.001 vs. control). **B**. Differences in wound closure rate over time between incubation in control medium (0.1% FBS) and positive control medium (10% FBS). Points represent mean wound closure rate + SEM of triplicate experiments (**p < 0.01; ***p < 0.001 vs control). **C**. Effect of addition of various concentrations of bronchoalveolar lavage fluid (BALF) from a term newborn diluted in control medium (0.1% FBS) on *in vitro *alveolar epithelial wound healing (t = 24 hours). Bars represent mean wound closure + SEM (% of control) of triplicate experiments (*p < 0.05; **p < 0.01 vs. control). **D**. Effects of various concentrations of albumin and BALF (diluted 1:10) from a term newborn (total protein in the original BALF specimen was 1.7 mg/ml). Bars represent mean wound closure + SEM relative to control medium of triplicate experiments (***p < 0.001 vs. control).

The effect on wound healing of addition of increasing FBS concentrations to culture medium was evaluated in order to validate the model (Figure 1A). Addition of FBS concentration-dependently stimulated wound closure. A large number of cells detached from the culture plate during incubation without FBS over 24 hours, while cell attachment was conserved and partial wound closure occurred during incubation in 0.1% FBS. Thus, this concentration was used as the optimal concentration to preserve cell viability and determine the effect of subsequent addition of BALF.

### Wound closure rate

To evaluate wound closure rate, wounded monolayers were incubated in control medium (0.1% FBS (v/v)) and positive control medium (10% FBS (v/v)) with serial photographs taken over a 24-hour period (Figure 1B). In control medium, wound closure rate decreased over time, stabilising between four and six hours of incubation. A more gradual decrease was observed during incubation with positive control medium.

### BALF has a concentration-dependent effect on wound closure

To evaluate the optimal dilution factor for BALF addition to the model, and evaluate the concentration dependency of the BALF effect on wound healing, BALF obtained from a term newborn was added in various concentrations to the model (Figure 1C). Addition of BALF to control medium had a concentration-dependent stimulatory effect on wound closure. For further experiments BALF was diluted 1:10.

### Albumin does not affect wound closure

Albumin is the most abundant protein in BALF and may be increased in serious lung injury as a result of distortion of the blood-air barrier. We evaluated the effect of addition of albumin to control medium on wound closure in order to determine whether this would affect our model (Figure 1D). Albumin did not significantly affect wound closure, while wound closure was significantly enhanced by addition of BALF, which contained less protein than the highest albumin concentration used in the experiment.

### BALF characteristics and wound healing

74 BALF specimens were collected from 45 ventilated preterm infants. There was no significant difference in total protein or wound closure between different postnatal days when all patients were considered (not shown). No significant differences in gestational age or birth weight were present between the different patient groups (Table 1). Patients with chorioamnionitis had significantly increased BALF total protein at day 7 when compared to patients without chorioamnionitis. No other differences in total protein were observed at any postnatal day. Wound closure correlated significantly with BALF total protein content (R^2 ^= 0.44; p < 0.001) and wound closure data are corrected for total BALF protein content in further analyses. Neither wound closure nor BALF total protein correlated with either gestational age or birth weight. There was a close correlation between wound closure at t = 6 and t = 24 (R^2 ^= 0.80; p < 0.001), yet the majority of the effect was reached within 6 hours after wound induction. Further analyses were performed only at t = 6.

**Table 1:** Patient characteristics.

Wound healing experimental group	Chorio (n = 7)	No chorio (n = 30)	Steroids (n = 35)	No steroids (n = 10)
Gestational age (weeks) (median (IQR))	26.9 (25.6-28.7)	28.6 (27.0-39.3)	28.6 (26.9-29.4)	27.5 (26.2-29.7)

Birth weight (grams) (mean ± SD)	1027 ± 246	1069 ± 221	1091 ± 223	1028 ± 268

BALF total protein day 0/1 (mg/ml) (mean ± SD)	0.75 ± 0.49 (n = 7)	0.76 ± 0.47 (n = 28)	0.82 ± 0.57 (n = 32)	0.62 ± 0.46 (n = 9)

BALF total protein day 3/4 (mg/ml) (mean ± SD)	1.47 ± 2.38 (n = 5)	1.25 ± 1.34 (n = 14)	1.09 ± 1.22 (n = 19)	3.21 ± 3.53 (n = 2)

BALF total protein day 7 (mg/ml) (mean ± SD)	0.84 ± 0.39* (n = 3)	0.44 ± 0.18 (n = 7)	0.51 ± 0.28 (n = 9)	0.95 ± 0.03 (n = 2)

**Total population**	**Chorio** **(n = 8)**	**No chorio** **(n = 37)**	**Steroids** **(n = 51)**	**No steroids** **(n = 11)**

Gestational age (weeks) (median (IQR))	26.5 (25.7-28.4)	28.4 (26.8-29.4)	28.6 (26.9-29.4)	27.6 (26.3-29.7)

Birth weight (grams) (mean ± SD)	1014 ± 231	1045 ± 228	1079 ± 236	1045 ± 260

General characteristics of different patient groups included in the cell culture experiments and the total population (including VEGF and KGF quantification).

*p < 0.05 vs no chorioamnionitis day 7.

Abbreviations: chorio = chorioamnionitis; IQR = interquartile range; SD = standard deviation.

To evaluate the effects of chorioamnionitis and antenatal steroids, the capacity of BALF to stimulate alveolar epithelial repair *in vitro *was compared between infants with and without chorioamnionitis and infants that had and had not received antenatal steroids.

### Chorioamnionitis decreases wound healing capacity of BALF

**Figure 2:**
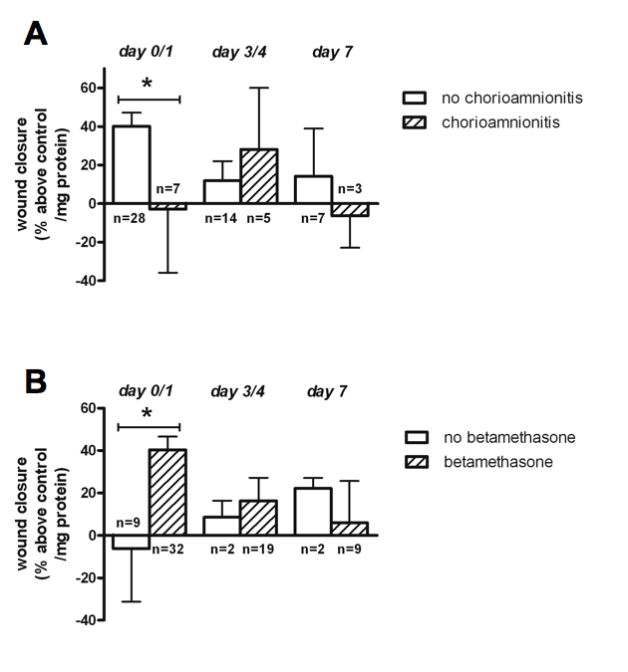
**Effects of chorioamnionitis and antenatal steroids on bronchoalveolar lavage fluid (BALF) wound healing capacity**. Effect of BALF obtained at different postnatal days from patients with and without chorioamnionitis (**A**), and from patients with and without antenatal steroid exposure (**B**) on *in vitro *alveolar epithelial wound healing. Bars represent mean wound closure +/- SEM (% above control/mg total protein in BALF) of triplicate experiments (*p < 0.05).

BALF obtained from infants with histological chorioamnionitis demonstrated a significantly decreased capacity to stimulate *in vitro *wound healing when compared to BALF from infants without histological chorioamnionitis at day 0/1 (Figure 2A). No significant differences were observed between infants with and without chorioamnionitis on postnatal days 3/4 and 7.

### Prenatal steroids stimulate wound healing capacity of BALF

BALF from infants exposed to antenatal steroids demonstrated a significantly increased capacity to stimulate wound healing at day 0/1 when compared to BALF from non-exposed infants (Figure 2B). No significant differences were observed after day 0/1, although it should be noted that at these time points the no steroids group comprised only two patients.

To further investigate the possible mechanisms of the effects of chorioamnionitis and antenatal steroids on the ability of BALF to repair alveolar epithelial injury in vitro, we focused on BALF-induced apoptosis and possible roles for KGF and VEGF, known modulators of lung development and lung injury repair. KGF and VEGF were quantified in BALF and added to the model in various concentrations.

### BALF-induced apoptosis is not influenced by chorioamnionitis or antenatal steroids

**Figure 3:**
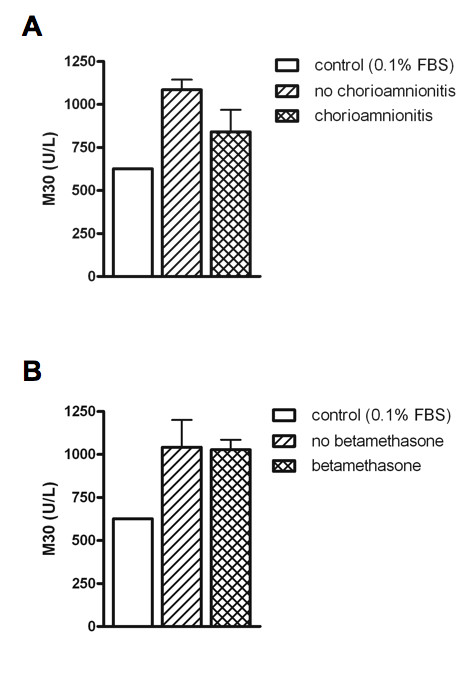
**M30 detected in culture supernatant after day 0/1 wound healing experiment according to chorioamnionitis or betamethasone exposure**. Bars represent mean wound closure + SEM per group. No significant differences between groups were present.

To investigate whether the effects of chorioamnionitis and antenatal steroids on day 0/1 could be explained by differential effects on BALF-induced apoptosis in A549 cells, we quantified M30, a soluble marker of epithelial apoptosis, in the cell culture supernatant at the end of the wound healing experiment. No significant differences were observed between culture supernatants of day 0/1 experiments from infants with or without chorioamnionitis (841 ± 338 vs. 1085 ± 316 U/L, p = 0.08; Figure 3A), and from infants exposed or not exposed to antenatal betamethasone (1028 ± 314 vs. 1041 ± 389 U/L, p = 0.42; Figure 3B).

### Chorioamnionitis is associated with decreased KGF and less detectable VEGF in BALF

KGF and VEGF were quantified in 121 BALF specimens from 62 patients, including all BALF specimens tested in the *in vitro *wound healing experiments. KGF was detected in 63%, 53%, 71% and 56% of BALF specimens obtained on day 0, 1, 3/4 and 7, respectively. KGF in BALF correlated positively with gestational age on day 0, but not thereafter (R^2 ^= 0.17, p < 0.05). VEGF was detected in 88%, 97%, 94% and 100% of BALF specimens obtained on day 0, 1, 3/4 and 7, respectively.

**Figure 4:**
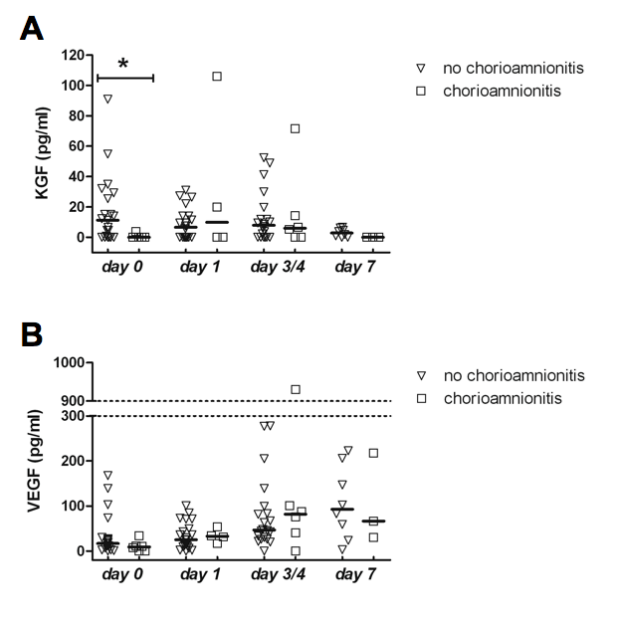
**Keratinocyte growth factor (KGF) and vascular endothelial growth factor (VEGF) levels in bronchoalveolar lavage fluid (BALF)**. KGF (**A**) and VEGF (**B**) levels in bronchoalveolar lavage fluid (BALF) obtained at different postnatal days from patients with and without chorioamnionitis. Points represent individual BALF growth factor concentrations, horizontal lines represent medians (*p < 0.05).

KGF concentrations in BALF obtained on day 0 from patients with chorioamnionitis were significantly lower than those in BALF from patients without chorioamnionitis (0 (0-5) vs. 11 (0-27), p < .05; Figure 4A). Furthermore, no KGF was detectable in chorioamnionits-exposed infants at day 7, while 75% of non-exposed infants had detectable KGF (p < 0.05). BALF from infants with chorioamnionitis was significantly less likely to contain detectable levels of VEGF on day 0 when compared to BALF from infants without chorioamnionitis (67% versus 95%, p < 0.05). Absolute VEGF concentrations were not significantly different between infants with or without chorioamnionitis on day 0 (9 (0-17) vs. 17 (7-29), p = 0.14), or on any other day (Figure 4B).

Concentrations of both KGF and VEGF in BALF correlated significantly with the ability of BALF to stimulate alveolar epithelial repair *in vitro *albeit with low R^2 ^values (R^2 ^= 0.08, p < 0.05; and R^2 ^= 0.10, p < 0.01, respectively). This illustrates that small part of the decreased ability of BALF obtained from patients with chorioamnionitis to stimulate alveolar epithelial repair may result from the decreased availability of these growth factors. No significant differences in BALF VEGF or KGF concentrations were detected on any day between patients that had or had not received antenatal steroids.

### KGF, VEGF and *in vitro *wound healing

**Figure 5:**
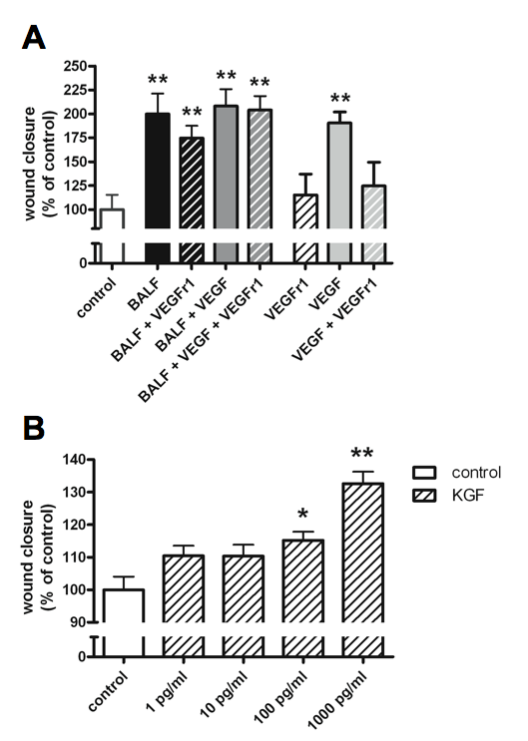
**Vascular endothelial growth factor (VEGF) and keratinocyte growth factor (KGF) effects on alveolar epithelial wound repair**. Effect of addition of different combinations of bronchoalveolar lavage fluid (BALF), VEGF (10 pg/ml), and soluble VEGF-receptor 1 (sVEGFr1; 6 ng/ml) (**A**), or various concentrations of KGF (**B**) on *in vitro *alveolar epithelial wound healing. Bars represent mean wound closure + SEM (% of control) of triplicate experiments (*p < 0.05, and **p < 0.01 vs control). No significant differences are present between BALF alone and BALF with any addition. Significant differences between VEGF, and both sVEGFr1 and VEGF + sVEGFr1 are present (both p < .05).

Remaining BALF was pooled for further experiments aimed at evaluating the roles of KGF and VEGF in BALF on wound repair. The pooled BALF contained 40 pg/ml VEGF, but undetectable levels of KGF. In the absence of BALF, VEGF stimulated alveolar epithelial repair, an effect that was blocked by addition of sVEGFr1 (Figure 5A). However, addition of the same concentrations of sVEGFr1, VEGF, or a combination of both to BALF did not significantly alter its effect on wound healing (Figure 5A). To evaluate whether natural abundance of sVEGFr1 in BALF could explain the inability to detect a VEGF-mediated effect on BALF-induced alveolar epithelial wound repair, we quantified sVEGFr1 in a subset of remaining BALF samples (n = 10). sVEGFr1 was present in significant amounts in all evaluated samples (median [range] = 5.39 [1.28; >10] ng/l).

Given the undetectable levels of KGF in the BALF pool, no attempts to antagonise the effect of KGF in BALF were made. Nevertheless, KGF was shown to have a concentration dependent stimulatory effect on wound healing (Figure 5B).

## Discussion

We report the development and characterisation of a translational *in vivo/in vitro *model for alveolar epithelial repair. Wound repair was quantified after mechanical wounding of a monolayer of A549 cells. FBS and BALF stimulated alveolar epithelial wound healing in this model in a concentration-dependent manner. Physiological concentrations of albumin, the most abundant protein in BALF, did not affect wound repair indicating that the effect of BALF results from actions of specific mediators rather than a general protein or dilutional effect. BALF from patients with chorioamnionitis had a significantly reduced capacity to induce alveolar epithelial wound healing, while exposure to antenatal steroids was associated with increased wound healing capacity of BALF shortly after birth. These effects were not attributable to alterations in BALF-induced apoptosis. BALF from patients exposed to chorioamnionitis contained lower levels of KGF and less detectable VEGF on postnatal day 0. BALF wound healing capacity correlated with levels of both of these and addition of these factors to our model resulted in stimulation of alveolar epithelial repair. However, results from subsequent experiments suggest that, at the concentrations present, their contribution to BALF-induced wound repair is at best around 10%, although significant effects could not be demonstrated.

Evaluation of epithelial wound healing *in vitro *is an established model for epithelial repair after injury and has been applied and characterised for many different epithelial types, including the alveolar epithelium [8]. Many investigators have used primary cultures of alveolar type II cells isolated from animal lungs in epithelial repair models since A549 cells do not hold all characteristics of type II cells and freshly isolated human type II cells are not readily available [8,27,29]. Still, of all human cell-lines available A549 cells share most characteristics with alveolar type II cells and are most widely used in human *in vitro *models of alveolar physiology. More importantly, in testing the biological activity of human BALF, we refrained from using animal-derived type II cells because of concerns regarding loss or modulation of biological signal due to species difference, in accordance with previous reports [9,10].

We are aware of only two earlier reports evaluating the effect of human lung-derived fluid on alveolar epithelial repair *in vitro *[9,10]. Perkins et al. recently showed that in patients ventilated for acute respiratory distress syndrome (ARDS), treatment with salbutamol, a β_2_-agonist, significantly increased the ability of BALF to stimulate wound healing in A549-cells [10]. Interestingly, BALF derived from salbutamol-treated patients also contained higher levels of VEGF, although the authors show evidence that in their study interleukin-1β (IL-1β) and not VEGF was the main contributor to epithelial repair [10]. In an earlier report, pulmonary edema fluid from ventilated adult patients with acute lung injury (ALI)/ARDS enhanced wound repair when compared to plasma or edema fluid from ventilated patients with hydrostatic edema [9]. Additional experiments suggested that IL-1β accounted for part of this effect, although edema fluid IL-1β concentrations were not reported. IL-1β was not evaluated in our model since BALF levels were increased rather than decreased after chorioamnionitis in our cohort (unpublished observations), arguing against its contribution to the decreased wound healing ability of BALF. IL-1 receptor antagonist (IL-1ra) induced by BALF in A549 cells or present in the alveolar space may further modulate the effects of IL-1β, complicating assessment of its contribution to our findings. Considering the inflammatory nature of both ALI/ARDS and chorioamnionitis, one might expect that these conditions have similar effects on wound repair. However, chorioamnionitis was associated with decreased wound healing in our model whereas BALF from ALI/ARDS patients increased wound repair [9]. Total protein concentrations in edema fluid from ALI/ARDS patients were also significantly higher [9], possibly explaining part of its increased wound healing potential. Furthermore age-related differences, differences in specimen used (BALF versus edema fluid) and growth factor patterns may partly account for differences.

A unique aspect of our study is the evaluation of the differential effect of BALF collected consecutively within distinct patient groups. We observed clear effects of the antenatal antecedents studied on wound healing capacity of BALF early after birth, whereas no significant effects were present later on. Thus, the effects of modulators of wound healing present in BALF on epithelial repair appear to vary over time. Multiple studies have shown that short term exposure to injury may induce important and sustained lung damage [30,31]. Therefore, modulation of lung injury repair by antenatal events seen in our study is likely to be of clinical importance, even though the effects are only observed shortly after birth. A larger time window between the antecedent and its effect, and possible interference of other factors influencing repair capacity may explain disappearance of the association over time [32].

The decreased capacity of BALF from patients previously exposed to chorioamnionitis to restore epithelial integrity provides a novel link between antenatal inflammation and adverse lung development [3,4,33]. Our model is unique in comparing biological activity of human BALF between patients with and without chorioamnionitis. The data suggest that prenatal exposure to inflammation results in BALF that is less efficient in repairing lung injury. This may explain why chorioamnionitis alone may not predispose to adverse lung development, while additional exposure to postnatal injurious factors such as mechanical ventilation and sepsis highly augments its association with chronic lung disease [11,12]. Experimental chorioamnionitis in preterm sheep increases the inflammatory response to postnatal mechanical ventilation, suggesting that inflammatory factors play a role [34]. Paradoxically, classic inflammatory cytokines known to be upregulated in chorioamnionitis have previously been shown to stimulate wound repair [8,9]. The finding of decreased epithelial repair after chorioamnionitis in our model suggests an additional role for other modulators. Although our initial experiments suggest that decreased levels of VEGF and KGF after chorioamnionitis may explain part of the decreased ability of BALF to induce alveolar epithelial repair, a definite contribution of neither factor could be established. We were able confirm earlier reports of stimulatory effects of VEGF and KGF on *in vitro *alveolar epithelial wound healing [27-29], and decreased availability of VEGF after chorioamnionitis [7]. Moreover, both KGF and VEGF levels in BALF are linked to development of BPD in previous reports [16-18,26], as well as in our sample [15]. A considerable amount of animal experimental data further supports a pivotal role of both factors in lung development and repair [19-25,27,28,35]. Thus, the early decrease in BALF levels of KGF and VEGF is likely to contribute to adverse lung development seen after chorioamnionitis, although our experiments suggest that their effects are primarily exerted through mechanisms other than BALF-induced alveolar epithelial repair. The overall low levels of VEGF and KGF as compared to adult studies may reflect developmental regulation as well as differences in BAL technique (e.g. non-bronchoscopic versus bronchoscopic). Furthermore, the potential role of the apparent abundance of sVEGFr1 in the alveolar space in decreasing VEGF bioavailability warrants further investigation [36].

Although antenatal steroids reduce RDS incidence, enhance structural maturity of the lung and improve lung function [1,2,37], BPD incidence seems unaffected [1]. However, when only studies using betamethasone are considered, BPD is significantly reduced [1]. By demonstrating an association between antenatal betamethasone and enhanced alveolar epithelial repair, our data provide a potential link between betamethasone exposure and improved lung development.

An earlier study found no effect of corticosteroids on airway epithelial repair [38], while another showed dexamethasone to inhibit wound repair initially, while inducing extended subsequent wound-healing potential in bronchial epithelial cells [39]. Thus, the direct effect induced by corticosteriods seems to differ from the longer term changes that affect the cellular response to injury. In sheep, maternal betamethasone acetate administration results in peak fetal plasma concentrations after 3 hours, dropping below the detection limit within 8 hours [40]. Since infants in our study were exposed to maternal steroids hours to weeks before birth, indirect mechanisms most likely explain the increased BALF wound healing capacity seen in our model. We were unable to detect betamethasone effects on BALF-induced apoptosis, and VEGF or KGF levels, suggesting that other factors play a role in this process.

Our model obviously is a simplified representation of the *in vivo *situation, since the alveolar space consist of a combination of different cell types. Communication between these cells cannot be investigated in our current model. Still we feel this model is an important first step in the identification of risk factors and protective factors in lung injury repair and of the underlying mechanisms at a molecular level. Larger patient groups are needed to further evaluate possible other modulating factors, such as oxygen exposure, mechanical ventilation characteristics and the combined effects of chorioamnionitis and antenatal steroids [3,41].

## Conclusion

A translational *in vivo/in vitro *model for alveolar epithelial repair using BALF as a reflection of the *in vivo *pulmonary environment, was characterised and applied. The evaluation of alveolar epithelial repair capacity of BALF serially collected in distinct patient groups provides a unique means by which biological activity of human specimens can be linked to clinical parameters over time. BALF obtained shortly after birth from preterm infants exposed to chorioamnionitis had a significantly decreased ability to restore alveolar epithelial integrity *in vitro*, providing a biological link between chorioamnionitis and adverse lung development. Our data suggest that the contribution of VEGF and KGF in BALF to this effect is probably small, although their decrease after chorioamnionitis may well modulate the susceptibility for adverse lung development. Conversely, prenatal steroid administration was shown to significantly increase wound-healing capacity of BALF in preterm infants. Additional research is needed to study the underlying mechanisms of modulation of alveolar epithelial repair by chorioamnionitis and antenatal steroids. Further evaluation of biological activity of human specimens in translational models like the one presented here carries great potential to study mechanisms underlying disease associations.

## Abbreviations

ALI: acute lung injury; ARDS: acute respiratory distress syndrome; BALF: bronchoalveolar lavage; BPD: bronchopulmonary dysplasia; FBS: fetal bovine serum; IL-1β: interleukin-1β; KGF: keratinocyte growth factor; L-glut: L-glutamin; P/S: penicillin/streptomycin; PBS: phosphate-buffered saline; RDS: respiratory distress syndrome; sVEGFr1: soluble VEGF-receptor 1; TAF: tracheal aspirate fluid; VEGF: vascular endothelial growth factor.

## Competing interests

The authors declare that they have no competing interests.

## Authors' contributions

JB designed the study, collected part of the patient samples, developed the cell culture model, carried out laboratory and statistical analyses, and drafted the manuscript. LZ designed the study, and supervised the study, analyses and manuscript preparation. AD collected the majority of the patient samples and aided in manuscript preparation. NK developed the cell culture model, carried out laboratory analyses, and aided in manuscript preparation. FvI designed the study, and supervised the study, laboratory work, further analyses and manuscript preparation.
